# Insulin-like growth factor-1 on cycle day 2 and assisted reproductive techniques outcome: A cross-sectional study

**DOI:** 10.18502/ijrm.v21i2.12804

**Published:** 2023-03-08

**Authors:** Fariba Abbasi, Robab Davar, Farimah Shamsi, Saeideh Dashti

**Affiliations:** ^1^Research and Clinical Center for Infertility, Yazd Reproductive Sciences Institute, Shahid Sadoughi University of Medical Sciences, Yazd, Iran.; ^2^Center for Healthcare Data Modeling, Department of Biostatistics and Epidemiology, School of Public Health, Shahid Sadoughi University of Medical Sciences, Yazd, Iran.

**Keywords:** Insulin-like growth factor-1, Poor ovarian response, Oocyte retrieval, Assisted reproductive techniques outcome.

## Abstract

**Background:**

Individualized assisted reproductive techniques (ART) can improve ART outcomes. Some studies suggested using insulin-like growth factor-1 (IGF-1) level on cycle day 2 for individualized ART.

**Objective:**

To investigate the relationship between serum levels of IGF-1 on day 2 of the cycle and ART outcomes.

**Materials and Methods:**

In this cross-sectional study, cycle day 2 serum levels of IGF-1 were measured in 175 women aged between 18-44 yr as candidates for in vitro fertilization or intracytoplasmic sperm injection. All participants received antagonist protocol, and the relationship between serum levels of IGF-1 and ART outcomes according to the number of oocytes were investigated; poor responders (oocytes < 5), normal responders (oocytes 5-15), and hyper responders (oocytes > 15).

**Results:**

Poor responders had higher serum level of IGF-1 when compared with normal and hyper-responders; however,this difference was not statistically significant (p = 0.41). The serum levels of IGF-1 in women with zero retrieved oocytes and those cycles that were canceled for the inappropriate ovarian response were not significantly different compared to other women in the group of poor responders. An inverse relationship was observed between the serum level of IGF-1 and anti-Mullerian hormone. Furthermore, no significant relationship between serum level of IGF-1 with age, body mass index, number of 2 pronucleus, and number of embryos was observed.

**Conclusion:**

According to our results, the serum levels of IGF-1 may not be able to predict ART outcomes. It seems necessary to conduct more studies with larger sample size.

## 1. Introduction

10-15% of couples suffer from infertility. Many of these patients need assisted reproductive techniques (ART) (1). One of the suggestions to improve ART outcomes, especially in poor responders (5-35% of ART cycles) (2-4), is individualized ovarian stimulation (5, 6) that needs efficient and reliable indicators. An ideal dynamic indicator shows the effect of pretreatment and adjuvant therapy on the metabolic environment of oocytes and can improve ART outcomes (7).

Currently, indicators such as anti-Mullerian hormone (AMH), follicle-stimulating hormone (FSH), antral follicle count, and the dynamic tests of the ovarian reserve like clomiphene citrate challenge test, are used for individualized ovarian stimulation (8). The high cost and low efficiency of these indicators, especially their inability to show the effect of pretreatments on ART outcomes, lead to a search for more cost-effective indicators. The insulin-like growth factor-1 (IGF-1) is one of the subjects that has the attention of researchers for its role in oocyte growth and embryo quality (9-11). IGF-1 is a single-chain polypeptide that is produced in the liver in response to growth hormone and transported to the organs, which mediates growth hormone effects (8). This factor can induce granulosa cell proliferation and on the other hand, FSH itself can facilitate granulosa cell synthesis of IGF-1 it regulates the entrance of follicles from the gonadotropin-independent stage to the gonadotropin-dependent stage. Also, IGF-1 stimulates folliclular growth and oocyte maturation by increasing the aromatase enzyme activity and the production of estrogen, progesterone, and the expression of the LH receptor (12-15).

Studies show that IGF-1 effectively protects follicular reserve and smaller antral follicles (16). IGF-1 increases FSH actions in granulosa cells in rodents and also low IGF levels in the follicular fluid lead to decreased response to FSH in in vitro fertilization (IVF). In addition, IGF-1 receptor inactivation has been associated with ovulatory dysfunction and infertility (17, 18), and lower levels of IGF-1 in follicular fluid are associated with low-quality oocytes and embryos (19-21). Increasing IGF-1 in follicular fluid in women > 35 yr old leads to improved cycle outcomes (22). Studies have shown that level of IGF-1 on the day 2 of the menstrual cycle has an inverse relationship with the ovarian response. And the IGF-1 serum level was found to be significantly higher in poor responders compared to women with normal and hyper responses. Decreased IGF-1 levels after estradiol pretreatment at the end of the late follicular phase improves ART outcomes (7, 23). Now the question arises is, can IGF-1 have a predictive value to individualize ovarian stimulation in ART cycles.

The present study was designed to investigate the relationship between serum levels of IGF-1 on the day 2 of cycle and ART outcomes.

## 2. Materials and Methods

This cross-sectional study was conducted at Yazd Reproductive Sciences Institute, Yazd, Iran from December 2021 to July 2022.

175 infertile women aged between 18-44 yr with body mass index (BMI) of 18-30 kg/m^2^ were considered as candidates for IVF or intracytoplasmic sperm injection (ICSI) with the antagonist protocol. All women who had used any pretreatment like estradiol, had any medical diseases, or endometriosis were excluded from this study.

Serum IGF-1 levels in all women were measured on cycle day 2; all the women received antagonist protocol. Gonadotropins ([Cinnal-f, CinnaGen, Iran] or [Homegnan, Daroupakhsh, Iran]) were started on day 2 of the menstrual cycle. The gonadotropin type and dose were adjusted for each woman based on age, AMH level, and antral follicle count. Gonadotropin-releasing hormone antagonist 0.25 mg (Cetrorelix, Ronak pharmamaceutical Co., Iran) was started when the dominant follicle reached 13-14 mm. The human chorionic gonadotropin (Gonarex, Ronak Darou) and/or gonadotropin-releasing hormone agonist (Triptorelin, Variopeptyl 0.1 mg, Varian Pharmed), were used for the final oocyte maturation when at least 2 lead follicles reached a mean diameter ≥ 17 mm. Ultrasound-guided transvaginal oocyte retrieval was performed 36 hr after final oocyte maturation. Vaginal progesterone suppository (Actogest, AtiPharmed, Iran) was administered from retrieval day. 18-24 hr after IVF or ICSI, fertilization was checked by observing 2 nucleated (2PN) cells. The fresh transfer was performed 48-72 hr after oocyte retrieval in eligible women. Embryo transfer was not performed in some women due to the participant's request, high progesterone levels on the trigger day, unsuitable endometrium, and risk of ovarian hyperstimulation syndrome.

The participants were divided into 3 groups according to the number of retrieved oocytes, the poor responders' group included women with oocytes < 5 or cycles were canceled due to inadequate response, the normal responders group (5-15 oocytes), and the hyper responders group ( > 15 oocytes).

Primary outcome include the relationship between cycle day 2 serum levels of IGF-1 and the number of retrieved oocytes. The fertilization rate and pregnancy rate per embryo transfer were considered as secondary outcomes. The fertilization rate was calculated as a percentage of 2PN to the number of mature oocytes and serum beta human chorionic gonadotropin titer > 50 IU/L, 14 days after the transfer was considered positive for chemical pregnancy.

### Sample size

Considering the confidence level of 95%, the power of 80%, and the sensitivity of 70% for IGF-1 according to a similar study and the loss of 10%, the minimum required for sample size was detected. The following formula was used: 


$$N=\frac{{\left(z1-\frac{\alpha }{2}+z1-\beta \right)}^{2}\times p\left(1-p\right)}{d^{2}}$$

### Ethical considerations 

The study was approved by the Ethics Committee of Yazd Reproductive Sciences Institute, Shahid Sadoughi University of Medical Sciences, Yazd, Iran (Code: IR.SSU.RSI.Rec.1400.014). Written informed consent was obtained from each participant after explaining the purpose of the study. All participants were assured of the confidentiality of the information obtained in the study.

### Statistical analysis

The SPSS software (Statistical Package for the Social Sciences, version 18.0, SPSS Inc., Chicago, Illinois, USA) was used for statistical data analysis. First, the normality of quantitative data was checked using the Kolmogorov-Smirnov test, and one-way ANOVA was used to check the mean of age and BMI in 3 groups. Kruskal-Wallis and Mann-Whitney tests were used to compare other quantitative variables between groups. The Chi-square test was used to compare the fertilization rate and the pregnancy rate per transfer. The Spearman correlation coefficient was used to check the correlation between IGF-1 and quantitative variables. P-value < 0.05 was considered statistically significant.

## 3. Results

Initially, 188 women were eligible to enter the study. Of them, 13 women were excluded due to the lack of information. Finally, the data from 175 women were analyzed and compared in 3 groups, the poor responders (n = 63), the normal responders (n = 82), and the hyper responders (n = 30).

The age in the poor responders was significantly higher than the normal and hyper responders; however, no significant difference was observed between the hyper and normal responders. AMH was found to be significantly lower in the poor responder group (Table I). The demographic characteristics of participants in 3 groups were reported in table I.

In poor responders, the cycle was canceled in 13 women due to inappropriate response to ovarian stimulation. No oocytes were obtained in 6 women and no embryo was formed in 11 participants. Embryo was not transferred in 7 women due to the participant's unwillingness and high progesterone levels, on the trigger day. Of 26 cases of embryo transfer 6 women had a positive pregnancy test. In normal responders, 8 women had high progesterone levels on trigger day, 10 women were planned to have PGD, 9 women withdrew from the embryo transfer due to personal reasons, and 15 cases had to freeze all the planning due to inappropriate endometrium. In 40 cases, embryos were transferred. The pregnancy test was positive in 10 cases. In the hyper responders group, embryo transfer was not done in 24 women due to the risk of ovarian hyperstimulation syndrome. Finally, in 6 cases embryo transfer was done, and in 2 cases, a positive pregnancy test was reported.

**Figure 1:**
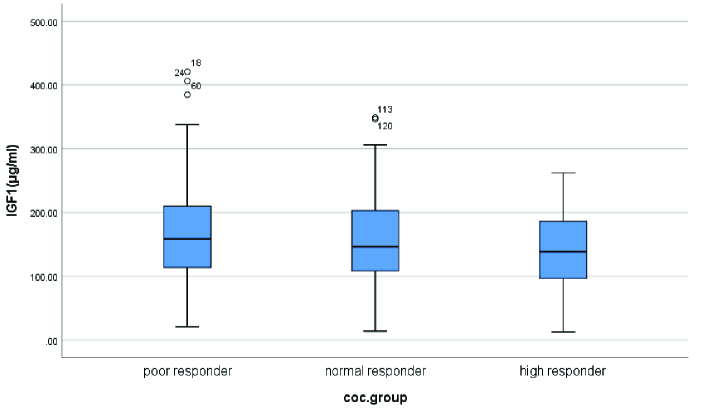
Comparison of cycle day 2 IGF-1 serum levels. COC: Cumulus oocyte complex, IGF-1: Insulin-like growth factor-1.

Cycle day 2 serum levels of IGF-1 in the poor responders group (169.57 ± 84.87) were higher than the normal responders (156.88 ± 75.01) and the hyper responders (141.44 ± 62.61). But this difference was not statistically significant (Figure 1). In the poor responders group, no significant difference was observed between the mean of cycle day 2 serum levels of IGF-1 in women with no retrieved oocytes (172.48 ± 97.62; Median: 162) and other participants in this group with 1-4 retrieved oocytes (168.31 ± 79.94; Median: 158.15) (p = 0.88).

IGF-1 serum level on day 2 of the cycle had an inverse relationship with AMH level (r = 0.17, p = 0.02). There were no correlations between cycle day 2 serum levels of IGF-1 and age, BMI, number of MII oocytes, total embryos, and fertilization rate (Table II).

**Table 1:** Comparison of demographic and cycle characteristics of the participants in 3 groups (n = 175)


**Characteristics**	**Poor responder group (n = 63)**	**Normal responder group (n = 82)**	**Hyper responder group (n = 30)**	**P-value**
Age (year)*	35.58 ± 5.08	32.91 ± 4.84	31.13 ± 5.50	< 0.001 ${}^{\text{a}}$
BMI (Kg/m^2^)*	26.50 ± 2.47	26.19 ± 3.54	25.88 ± 3.11	0.65 ${}^{\text{a}}$
AMH (ng/ml)*	1.06 ± 1.3 MD = 0.60, IQR = 1.1	2.99 ± 2.51 MD = 2.30, IQR = 2.42	6.67 ± 3.50 MD = 6.30, IQR = 5.37	< 0.001 ${}^{\text{b}}$
IGF-1 (µg/L)*	169.57 ± 84.87 MD = 158.50, IQR = 104.9	156.88 ± 75.01 MD = 146.35, IQR = 94.93	141.44 ± 62.61 MD = 138.45, IQR = 90.38	0.41 ${}^{\text{b}}$
Estradiol (pg/ml)*	778.54 ± 547.65 MD = 689.50, IQR = 854	1901.85 ± 1223.97 MD = 1610.00, IQR = 1344	4216.8 ± 2328.01 MD = 3473.00, IQR = 4098	< 0.001 ${}^{\text{b}}$
Progesterone (ng/ml)*	0.37 ± 0.80 MD = 0.20, IQR = 0.20	0.73 ± 0.98 MD = 0.50, IQR = 0.71	0.39 ± 0.65 MD = 0.10, IQR = 0.18	< 0.001 ${}^{\text{b}}$
LH (mIu/ml)*	3.73 ± 3.89 MD = 2.65, IQR = 3.05	2.68 ± 2.61 MD = 2.10, IQR = 2.99	2.78 ± 2.53 MD = 1.90, IQR = 3.95	0.22 ${}^{\text{b}}$
Mature oocytes*	1.96 ± 1.06 MD = 2.00, IQR = 2.00	6.96 ± 3.13 MD = 6.00, IQR = 5.00	14.76 ± 5.08 MD = 15.00, IQR = 8.00	< 0.001 ${}^{\text{b}}$
2PN*	1.16 ± 0.88 MD = 1.00, IQR = 1.00	4.32 ± 2.67 MD = 4.00, IQR = 4.00	10.03 ± 3.97 MD = 9.50, IQR = 6.00	< 0.001 ${}^{\text{b}}$
Total embryos (n)*	1.09 ± 0.91 MD = 1.00, IQR = 2.00	3.70 ± 2.54 MD = 3.00, IQR = 4.00	8.77 ± 3.83 MD = 8.50, IQR = 7.00	< 0.001 ${}^{\text{b}}$
Fertilization rate**	58.0% (51/88)	61.30% (346/564)	70.0% (301/430)	0.008 ${}^{\text{c}}$
Pregnancy rate per ET**	23.10% (6/26)	25% (10/40)	33.30% (2/6)	0.87 ${}^{\text{c}}$
*Data presented as Mean ± S.D. **Data presented as percentage (%). ${}^{\text{a}}$ One-way ANOVA test, ${}^{\text{b}}$ Kruskal-Wallis test, ${}^{\text{c}}$ Chi-square test, BMI: Body mass index, AMH: Anti-Mullerian hormone, IGF-1: Serum insulin-like growth factor-1, LH: Luteinizing hormone, 2PN: 2 pronucleus, ET: Embryo transfer, MD: Median, IQR: Interquartile range				

**Table 2:** Correlation between IGF-1 and other variables in participants


**Variable**	**Correlation coefficient**	**P-value**
**Age (Yr)**	-0.1	0.22
**BMI (Kg/m^2^)**	-0.12	0.12
**AMH (ng/ml)**	-0.17	0.02
**MII**	-0.05	0.52
**2PN**	-0.09	0.26
**Total embryos**	-0.07	0.37
BMI: Body mass index, AMH: Anti-Mullerian hormone, MII: Mature oocyte, 2PN: 2 pronucleus, Spearman Correlation Coefficient test		

## 4. Discussion

In this cross-sectional study, we investigated the relationship between the serum levels of IGF-1 on day 2 of the menstrual cycle and ART outcomes. Our results showed that serum levels of IGF-1 in the poor responders were higher than normal and hyper-responders but no significant difference was observed.

A retrospective study on 167 women, showed that the serum level of IGF-1 in poor responders is higher than in normal and hyper responders. Also, they found IGF-1 levels more than 70 ng/mg, with a sensitivity of 85 and a specificity of 80%, associated with a negative pregnancy result (23).

Another retrospective study on 184 cycles, showed that the IGF-1 level on day 2 of the cycle was significantly higher in women with poor ovarian response than in normal and hyper responders. Pretreatment with estradiol in the luteal phase decreased the serum level of IGF-1 and increased the number of retrieved oocytes and cumulative pregnancy rate. However, no effect on oocyte maturation rate and fertilization rate was seen. Also, they reported higher IGF-1 levels in women with canceled cycles where they concluded that an IGF-1 level of more than 72 ng/mg is a strong negative indicator for ART outcomes. By a receiver operating characteristic curve with 70% sensitivity and 78% specificity in poor responders, they suggested an IGF-1 level of more than 72 ng/mg as a threshold for delaying cycle until reducing the IGF-1 level with estradiol pretreatment (7).

Follicular resistance to IGF-1 can lead to an increased level of IGF-1 in poor responders (7). Ligand-dependent internalization of receptor has been suggested as the cause of resistance to IGF-1 (24). The present study showed an inverse relationship between the serum IGF-1 levels on day 2 of the cycle and AMH. While the level of IGF-1 was not related to BMI and age.

Yovich et al. reported no correlation between IGF profiles in the early follicular phase and AMH and antral follicle count (25). Another study reported an inverse relationship between age and serum level of IGF-1 (26). Considering the function of IGF-1 as a biochemical marker of follicular function, the use of IGF-I in follicular fluid as a potential marker of embryo quality and predictor of ART outcome was investigated (6, 21, 22).

Some studies showed that the level of IGF-1 in the follicular fluid is directly related to the embryo quality and the pregnancy rate (6, 21, 22). In women over 35 yr, the chance of pregnancy increases with the increase of IGF-1 in the follicular fluid. Treatment of poor ovarian responders is one of the most challenging topics in infertility. Using different ovarian stimulation protocols and increasing the dose and duration of stimulation with gonadotropins, in these patients, did not significantly improve ART outcomes (27, 28). Some research showed that increasing IGF-1 with growth hormone administration effectively improves ART outcomes in poor responders. Studies are being conducted to examine the possibility of prescribing growth hormones based on the serum level of IGF-1 (7, 19, 29, 30).

Reducing serum level of IGF-1 in poor responders by estradiol pretreatment has improved ART outcomes (7). In another way some researchers reported that pretreatment with estradiol in women with poor ovarian response did not increase the number of retrieved oocytes and fertilization rate (31). Finally, considering the conflicting results on the relationship between serum levels of IGF-1 with age, AMH level, and ART outcomes, it seems necessary to conduct more studies in this field.

## 5. Conclusion

According to our results, cycle day 2 serum levels of IGF-1 in the poor responder group were higher than the normal and the hyper responders; however, this difference was not statistically significant. The serum level of IGF-1 may not be able to predict ART outcomes. It seems necessary to conduct more studies.

##  Conflict of Interest

The authors declare that there is no conflict of interest. 
