# Correction to “Insulin-like growth factor-1 on cycle day 2 and assisted reproductive techniques outcome: A cross-sectional study” [Int J Reprod BioMed 2023; 21: 139-146]

**DOI:** 10.18502/ijrm.v22i8.17131

**Published:** 2024-10-14

**Authors:** Fariba Abbasi, Robab Davar, Farimah Shamsi, Saeideh Dashti

The publisher has been informed of an error that occurred on page 139 (Vol. 21; No. 2), which the ORCID ID of the corresponding author must be changed to “0000-0001-7089-0772”. The publisher wishes to apologize for this error. The online version of the article has been updated on August 31, 2024 and can be found at https://doi.org/10.18502/ijrm.v21i2.12804.

